# Heavy social media use and psychological distress among adolescents: the moderating role of sex, age, and parental support

**DOI:** 10.3389/fpubh.2023.1190390

**Published:** 2023-06-15

**Authors:** Fatima Mougharbel, Jean-Philippe Chaput, Hugues Sampasa-Kanyinga, Hayley A. Hamilton, Ian Colman, Scott T. Leatherdale, Gary S. Goldfield

**Affiliations:** ^1^School of Population Health, University of Ottawa, Ottawa, ON, Canada; ^2^Healthy Active Living and Obesity Research Group, Children's Hospital of Eastern Ontario Research Institute, Ottawa, ON, Canada; ^3^School of Epidemiology and Public Health, University of Ottawa, Ottawa, ON, Canada; ^4^Center for Addiction and Mental Health, Institute for Mental Health Policy Research, Toronto, ON, Canada; ^5^Center for Fertility and Health, Norwegian Institute of Public Health, Oslo, Norway; ^6^School of Public Health and Health Systems, University of Waterloo, Waterloo, ON, Canada

**Keywords:** social media, psychological distress, mental health, adolescents, screen time

## Abstract

**Background:**

Despite increasing evidence that social media use is associated with adolescents’ mental well-being, little is known about the role of various factors in modifying the effect of this association during adolescence. This study examined the association between social media use and psychological distress among adolescents and explored whether sex, age, and parental support moderate this association.

**Methods:**

Data came from a representative sample of middle and high school students in Ontario, Canada. Cross-sectional analyses included 6,822 students derived from the 2019 Ontario Student Drug Use and Health Survey.

**Results:**

Our results showed that 48% of adolescents used social media for 3 h or more per day, and 43.7% had moderate to severe psychological distress, with a higher prevalence among females (54%) than males (31%). After adjustment for relevant covariates, heavy social media use (≥3 h/day) was associated with increased odds of severe psychological distress [odds ratio (OR): 2.01; 95% confidence interval (CI):1.59–2.55]. The association of social media use with psychological distress was modified by age (*p* < 0.05) but not sex or parental support. The association was stronger among younger adolescents.

**Conclusion:**

Heavy social media use is associated with higher levels of psychological distress, with younger adolescents being the most vulnerable. Longitudinal studies are recommended for future research to examine in more depth the role of sex, age, and parental support in the association between social media use and psychological distress to better determine the strength and of the association.

## Background

Adolescence is a critical and formative transitory period in which individuals shift from childhood to adulthood. With significant biological, developmental, and psychological changes taking place during adolescence, it is crucial to ensure that adolescents are fully supported to promote physical and mental well-being to set the stage for a healthy adulthood (1, 2). During this period of life, adolescents become more independent in making choices related to their lifestyle behaviors (3). Early adolescence involves pubertal changes that elicit substantial biological, cognitive, physical, social, and emotional changes making this a critical period for the development of mental health problems (4, 5). Furthermore, the period of late adolescence through which adolescents are transitioning to adulthood, is also considered to be a highly vulnerable period for mental health problems that can have profound effects into adulthood (6). Accordingly, it is crucial to examine how modifiable behaviors during adolescence impact mental health outcomes, in order to address them before they become rooted.

Mental health problems among adolescents have increased worldwide, with one in seven (14%) young people aged 10–19 years experiencing mental health problems (1), within which anxiety and depression make up about 43% (2) and are considered the leading causes of illness and disability for adolescents (1). Depression and anxiety are also considered risk factors for many adverse health outcomes, such as cardiovascular diseases, behavioral problems, and substance use disorder (7, 8). In addition to that, depression and anxiety can affect academic achievement and learning, and reduce quality of life. At its worst, depression can lead to suicide (1). Recent evidence suggests that girls have worse mental health outcomes such as psychological distress than boys (9–11). With mental health morbidity increasing significantly among adolescents (12), underpinning factors are still unclear, but are likely complex and influenced by many environmental factors. Previous literature has suggested that increases in mental health problems may be associated with higher levels of social media use (13–16).

While the COVID-19 pandemic has accelerated the trend of social media use among adolescents when in-person interaction had been restricted (17–19), social media use had increased dramatically among adolescents and occupied a significant part of their daily lives even prior to the onset of the pandemic (20). Recently, the World Health Organization strongly recommended in their 2020 guidelines for physical activity and sedentary behavior that children and youth should limit the amount of recreational screen time (21). Furthermore, the Canadian 24-h movement guidelines for children and adolescents recommend limiting recreational screen time to 2 h or less for children and youth aged 5–17 years (22, 23). However, with the emergence of newer devices such as tablets and smartphones, social media was considered as one of the most used screen activities among youth, especially females (24, 25). In Canada, 86% of youth in Ontario use social media platforms every day, and more than 20% indicate they spend more than 5 h on social media daily (26). This raises concerns given that adolescence, especially at its onset, is considered a crucial time frame because it is the period at which there is an exponential increase in social media use (27) and increasing vulnerability to mental health problems (4). Accordingly, understanding the association between social media use and adolescents’ mental health has become a priority.

Previous studies on social media use and adolescents’ mental health provides some evidence regarding the nature of the association (13, 14, 16, 24, 28–31). However, results were mixed, resulting in an inconclusive understanding to date. Inconsistent findings could be attributed to many reasons, such as differences in research designs, populations sampled, and analytical plans. Also, while various factors of social media use and mental health have been identified in previous research, most studies failed to examine whether this association differs by characteristics of individuals (16, 24, 32–34). For example, some studies reported sex differences in the association between social media use and mental health or performed stratified analyses by sex without statistically examining whether sex moderated the association (35–39), and in the few that considered the moderating role of sex on the relationship (13, 40, 41), results were inconsistent. Age has also been proposed as a factor that could modify the effect of social media use on mental health problems, yet, only one study examined the moderating role of age, and found that high social media use was associated with higher odds of internalizing problems among younger adolescents (42). Given the importance of the social media context during early adolescence, a period in which individuals give more values to peer acceptance, friendships, and identity exploration, early adolescents can be more vulnerable to harmful contents of social media (43, 44). In addition, recent research showed that females who use social media extensively at early ages had poorer mental health outcomes several years on (41). This suggests further examination of the role of age and sex in the association between social media use and mental health among adolescents (15, 16, 24).

In addition to the role of sex and age, research needs to better understand other factors such as the social and psychological support factors represented by the role of parents (24, 45). It has been suggested that weak parental supportiveness makes adolescents feel disconnected, so they use social media more frequently to find meaningful social interaction (45). However, very little is known about how parental support plays a role in social media use and adolescent depression and anxiety, and none of the studies examined the moderating role of parental support. Thus, considering the role of parents in this association is important and needed (24).

Since social media use is both highly prevalent among youth and a seemingly modifiable behavior, examining the variables that modify the effect of the association between social media use and psychological distress symptoms among youth is crucial to fill the knowledge gaps and inform future research and interventions. Accordingly, the objectives of this study were to (1) examine the association between social media use and psychological distress among adolescents, and (2) explore the moderating role of age, sex, and parental support on the association between social media use and psychological distress. We hypothesized that heavy social media use would be associated with psychological distress in adolescents. We also hypothesized that sex, age, and parental support would be moderators of this association with females, younger adolescents, and those who reported rare parental support being the most vulnerable for psychological distress when using social media heavily.

## Design

The current study is a secondary data analysis of the 2019 Ontario Student Drug Use and Health Survey (OSDUHS) (46). This representative cross-sectional school-based survey included Ontarians in grades 7–12 from English and French public and Catholic schools (*n* = 14,142). Two hundred sixty-three schools from 47 public and Catholic school boards participated in this survey. Ethics approval was obtained from the Research Ethics Boards of the Center for Addiction and Mental Health (CAMH; 029/2016), York University (e2014-099), and 47 public and Catholic school boards’ research review committees. Participation in the survey required active parental written consent and student assent. The survey was completed anonymously during school time.

Data were collected from November 2018 to June 2019. Four split ballot versions of the questionnaire were administered in classrooms in a paper booklet format (Form A and B for Elementary Schools; Form A and B for Secondary Schools) where students completed one of two alternately distributed questionnaires in classrooms depending on the school level. Questionnaires used in elementary schools excluded certain topics (e.g., drug use screeners for problematic drug use). Form A and Form B were distributed alternatively to students in each classroom to accomplish two near-equal random samples completing each form. The overall completion rate was 59%, with reasons for not completing the survey including unreturned consents or refusal of participation (29%), and student absenteeism (12%). Further information about the survey is available elsewhere (46). For the present study, only those who completed the questionnaire with the mental health component (Form A, *N* = 7,617) were included.

## Measures

### Social media use

Students were asked to indicate how many hours they spend daily posting or browsing social media sites, such as Facebook, Twitter, and Instagram. Response options were as follows: less than 1 h a day, about 1 h a day, 2 h a day, 3–4 h a day, 5–6 h a day, 7 or more hours a day, not daily usage, do not use social media, and do not use the Internet. We created a scale variable as follows: the three latter response options represented no use of social media (coded 0), the “less than 1 h a day,” “about 1 h a day,” and “2 h a day” responses were collapsed and represented light to moderate use (coded 1), the “3–4 h a day,” “5–6 h a day,” and “seven or more hours a day” were collapsed and represented heavy use (coded 2). The cut-off was specified based on the recommended cut-off of 2 h or less for daily recreational screen time among adolescents from the Canadian sedentary behavior guidelines (22) and previous studies on similar topics (47, 48).

### Psychological distress

The Kessler Psychological Distress Scale (K-6) was used to measure the frequency of experiencing depression and anxiety in the past 4 weeks (49). Each of the six items included the following response options: “none of the time,” “a little of the time,” “some of the time,” “most of the time,” and “all of the time.” A total score ranging from 0 to 24 was created, with higher scores representing greater psychological distress. A score ranging from 0 to 7 represented no distress (coded 0), 8 to 12 represented moderate distress (coded 1), and a score of 13 and more indicated severe distress (coded 2) (50), consistent with other studies using the K6 measure (51–53). The K6 is a valid and reliable tool to measure symptoms of psychological distress among youth with a high internal consistency (α = 0.86) (54). The internal reliability coefficient for the K-6 in this study was Cronbach’s α = 0.87.

### Potential moderators

Sex, age, and parental support were potential moderators in our study. Sex is represented by a binary variable: females (coded 1) and males (coded 0). Age was also represented by a binary variable and grouped as “younger adolescents” (10–14 years old, coded 0) and “older adolescents” (15–20 years old, coded 1) based on early adolescence and middle and late adolescence phases reported in the literature (55). Parental support was assessed by the following question: “How often do you talk about your problems and feelings with at least one of your parents?” The response options were: “always,” “usually,” “sometimes,” “rarely,” and “never.” We collapsed the first two categories into one category that represented good parental support (coded = 0). Responses of “sometimes” (coded = 1) remained a separate category, and the last two categories were collapsed to represent poor parental support (coded = 2).

### Covariates

Covariates included sex and age (if not moderators), ethnicity, subjective socioeconomic status (SES), and body mass index (BMI) *z*-score. Covariates were selected based on their availability in the dataset and their association with the independent and dependent variables (56–60). Ethnicity was measured through self-identification from the following categories: White, Chinese, Filipino, South East Asian, Japanese, Korean, South Asian, West Asian, Black, Aboriginal, Latino, and Other. We dichotomized this variable as follows: White (coded 0) in a separate category, and all multiple selections on the ethnicity groups included in another category (coded 1). SES was assessed using the MacArthur Subjective Socioeconomic Status Ladder (61). Students were asked to indicate on the 1–10 rungs ladder what best described their parents’ SES: “Imagine this ladder below shows how Canadian society is set up. At the top of the ladder are people who are the ‘best off’—they have the most money, the most education, and the jobs that bring the most respect. At the bottom are the people who are ‘worst off’—they have the least money, little education, no job or jobs that no one wants. Now think about your family. Please check off the numbered box that best shows where you think your family would be on this ladder.” Rungs higher on the ladder indicate higher perceived SES. This variable was treated as a continuous variable in our analyses. We also calculated BMI (km/m^2^) from students’ self-reported weight (in kilograms) and height (in meters), and we transformed it into *z*-scores following the reference data issued by the World Health Organization (62).

## Statistical analysis

In our analysis, we used Taylor series linearization methods to account for the complexity of the sample design of the survey and to attain unbiased variances and point estimates using Stata 16.1 (Stata Corporation, College Station, TX, United States). Analyses included complete information on all variables, reducing the sample size from 7,617 to 6,822. Pearson’s chi-square tests and adjusted Wald tests were used for categorical and continuous variables, respectively, to test the statistical differences between excluded data (missing data) and those included in our analyses for all the variables. Compared to the included participants, those who were excluded were more likely to be males, aged 15–20 years, less likely to be of white ethnicity, and more likely to use social media heavily.

Participant characteristics were described by proportions and means. Correlations between all variables of interest included in the study were examined using Spearman correlation for ordinal and continuous variables and Pearson chi-square adjusted for the survey design and transformed into an F-statistic for categorical variables because of the complex sampling. Ordinal logistic regression was used to estimate the odds ratios (OR) for the psychological distress outcome with all independent variables of interest. Furthermore, crude and adjusted ordered regression models were used to examine associations between social media use and psychological distress among adolescents. Covariates included age, sex, ethnicity, subjective SES, and BMI *z*-score. In order to test if the associations between social media and psychological distress varied by sex, age, and parental support, two-way interactions were examined in separate models. In cases where interaction terms were significant, stratified results were presented.

## Results

### Sample characteristics

Table 1 provides the descriptive characteristics of the sample. About 57% of the sample were females, 58% of the students were 15 years or older, and 56% were white. About 48% of the sample used social media heavily (≥3 h daily). The prevalence of moderate and severe psychological distress was 23.4 and 20.3%, respectively.

**Table 1:** Descriptive characteristics of the full sample and stratified by variables of interest.

Characteristics		Age		Sex		Parental support		
	Total sample	11–14 years	15–20 years	Males	Females	Always or usually	Sometimes	Rarely or never
	*N* = 6,822	*N* = 2,880	*N* = 3,942	*N* = 2,951	*N* = 3,871	*N* = 2,745	*N* = 1,667	*N* = 2,410
Sex								
Males	43.2	43.8	42.8			36.1	42.6	51.8
Females	56.8	56.2	57.2			63.9	57.4	48.2
Age								
11–14-year-old	42.2			42.8	41.8	45.5	41.4	39.0
15–20-year-old	57.8			57.2	58.2	54.5	58.6	61.0
Ethnicity								
White	55.5	55.0	56.0	55.0	56.5	60.0	54.4	51.3
Other	44.5	45.0	44.0	45.0	43.5	40.0	45.6	48.7
Subjective SES								
Mean	6.94	7.14	6.83	6.98	6.89	7.17	7.17	6.74
(SD)	(1.68)	(1.75)	(1.62)	(1.55)	(1.80)	(1.61)	(1.61)	(1.68)
BMI Z-score								
Mean	0.33	0.37	0.31	0.35	0.31	0.33	0.33	0.34
(SD)	(1.11)	(1.21)	(1.05)	(0.97)	(1.26)	(1.13)	(1.13)	(1.08)
Social media use								
No use	12.5	22.0	5.6	16.0	9.9	14.0	11.6	11.5
Light to moderate use (≤2 h/day)	39.5	39.3	39.8	46.0	34.6	43.6	40.2	34.5
Heavy use (≥3 h/day)	48.0	38.7	54.6	38.0	55.5	42.4	48.2	54.0
Parental support								
Always/usually	40.2	43.4	38.0	33.6	45.3	14.0	11.6	12.5
Sometimes	24.5	24.0	24.8	24.1	24.7	43.6	40.2	34.5
Rarely/never	35.3	32.6	37.2	42.3	30.0	42.4	48.2	54.0
Psychological distress								
No distress	56.3	64.2	50.4	69.9	46.0	68.1	54.5	44.0
Moderate distress	23.4	19.8	26.0	19.2	27.0	20.1	27.0	24.7
Severe distress	20.3	16.0	23.6	10.9	27.0	11.8	18.5	31.3

Data are shown as percentages or mean (standard deviation).

SES, socioeconomic status; SD, standard deviation; BMI, body mass index.

Among the younger students (11–14 years), 38.7% used social media heavily, 32.6% reported rare parental support, 19.8% were moderately distressed, and 16% were severely distressed. However, among older students, 54.6% reported using social media heavily, 37.2% reported rare parental support, 26% were moderately distressed, and 23.6% were severely distressed.

Regarding parental support, among those who reported having usual parental support (*N* = 2,745), 14% reported not using social media, 43.6% reported using social media moderately, and 42.4% reported heavy use of social media. Also, among the same group, 20.1% reported having moderate distress, and 11.8% reported having severe distress.

However, among those who reported having rare parental support (*N* = 2,410), 11.5% reported not using social media, 34.5% reported using social media moderately, and 54% reported heavy use of social media. In addition to that, of participants who reported having rare parental support, 24.7% reported having moderate distress, and 31.3% reported having severe distress.

### Bivariate analysis

Bivariate correlations for all variables of interest are shown in Table 2. The chi-square results indicate that being a male was correlated with lower psychological distress and less social media use. Being older (15–20-year-old) was correlated with higher psychological distress and more social media use. Non-white ethnicity was associated with higher psychological distress. Spearman correlation results indicate that higher SES was associated with lower psychological distress. Also, higher psychological distress was associated with more use of social media and less parental support. Furthermore, less parental support was associated with more social media use.

**Table 2:** Correlation results of the association between psychological distress, sociodemographic characteristics, and social media use among adolescents (*n* = 6,822).

	1.	2.	3.	4.	5.	6.	7.	8.
1. Sex	–							
2. Age	0.085^†^	–						
3. Ethnicity	1.021^†^	1.066^†^	–					
4. SES	0.018^‡^	−0.107^***‡^	−0.091^***‡^	–				
5. BMI z-score	−0.002^‡^	−0.005^‡^	0.003^‡^	−0.063^***‡^	–			
6. Social media use	57.40^***†^	95.019^***†^	9.408^***†^	−0.055^***‡^	0.039^***‡^	–		
7. Parental support	41.174^***†^	7.665^***†^	6.018^**†^	−0.189^***^	0.017^‡^	0.100^***‡^	–	
8. Psychological distress	179.200^***†^	30.906^***†^	1.833^†^	−0.223^**^*	0.055^***‡^	0.200^***‡^	0.232^***‡^	–

Sex (male/female), ethnicity (white/non-white), subjective SES, social media use (no use, moderate use, heavy use), parental support (usually, sometimes, rarely), and psychological distress (no distress, moderate distress, and severe distress).

SES, socioeconomic status; SD, standard deviation; BMI, body mass index.

†Pearson chi-square adjusted for the survey design and transformed into an F-statistic.

^‡^Spearman Correlation.

^*^p < 0.05; ^**^p < 0.01; ^***^p < 0.001.

### Multivariable ordinal regression modeling of the association between social media use and psychological distress among adolescents adjusted for all variables of interest

Results of multivariable ordinal regression analysis examining the association between social media use and psychological distress are summarized in Table 3. Heavy social media use was associated with psychological distress (OR: 1.44; 95% CI: 1.12–1.85). Also, adolescents who reported parental support was rare had higher odds of psychological distress compared to those with good parental support (OR: 2.41; 95% CI: 2.04–2.84). Adolescents who felt sometimes supported also had higher odds of psychological distress compared to those with good parental support (OR: 1.57; 95% CI: 1.31–1.87). Males were less likely than females to experience high psychological distress levels (OR: 0.41; 95% CI: 0.36–0.46).

**Table 3:** Results of the ordered regression analysis of the association between social media use and psychological distress adjusted for all variables of interest (*n* = 6,822).

Model	Psychological distress OR (95% CI)
Social media use	
No use	1
Light to moderate use (≤2 h/day)	1.11 (0.88–1.41)
Heavy use (≥3 h/day)	**1.44 (1.12–1.85)** ^**^
Sex	
Female	1
Male	**0.41 (0.36–0.46)** ^***^
Age	
11–14-year-old	1
15–20-year-old	**1.36 (1.18–1.58)** ^***^
SES	**0.83 (0.80–0.86)** ^***^
Race	
White	1
Non-white/ other	0.90 (0.78–1.05)
BMI *Z*-score	1.03 (0.97–1.08)
Parental support	
Usually/always	1
Sometimes	**1.57 (1.31–1.87)** ^***^
Rarely/never	**2.41 (2.04–2.84)** ^***^

Data are shown as odds ratio and 95% confidence interval. OR, odds ratio; CI, confidence interval. Model is adjusted for sex, age, subjective SES, ethnicity, BMI *z*-score, and parental support.

SES, socioeconomic status; SD, standard deviation; BMI, body mass index.

^*^p < 0.05; ^**^p < 0.01; ^***^p < 0.001. Bold values represent statistically significant results.

### Crude and adjusted multivariable ordinal regression modeling with interaction terms of the association of social media use on psychological distress among adolescents

Results of multivariable ordinal regression analysis examining the association between social media use and psychological distress with interaction terms are summarized in Table 4.

**Table 4:** Results of the crude and adjusted ordered regression analysis with interaction terms of the associations between social media use and psychological distress among adolescents (*n* = 6,822).

Models	Psychological distress	
	Crude	Adjusted’
Model 1		
No use	1	1
Light to moderate use (≤2 h/day)	1.21 (0.97–1.51)	**1.29 (1.02–1.62)** ^*^
Heavy use (≥ 3 h/day)	**2.28 (1.82–2.85)** ^***^	**2.01 (1.59–2.55)** ^***^
Model 2—Interaction term between social media use and sex		
Light to moderate use × sex	1.34 (0.87–2.06)	1.29 (0.82–2.03)
Heavy use social media use × sex	1.29 (0.86–1.93)	1.29 (0.85–1.95)
Model 3—Interaction term between social media use and age		
Light to moderate use × age	0.70 (0.43–1.15)	0.71 (0.43–1.16)
Heavy social media use × age	**0.56 (0.34–0.93)** ^*^	**0.54 (0.33–0.90)** ^*^
Model 4—Interaction term between social media use and parental support		
Light to moderate use × sometimes parental support	0.70 (0.36–1.32)	0.75 (0.38–1.50)
Light to moderate use × rare parental support	0.75 (0.43–1.29)	1.01 (0.58–1.77)
Heavy social media use × sometimes parental support	**0.51 (0.27–0.94)** ^*^	0.53 (0.27–1.03)
Heavy social media use × rare parental support	**0.53 (0.31–0.91)** ^*^	0.69 (0.39–1.21)

Data are shown as odds ratio and 95% confidence interval. OR, odds ratio; CI, confidence interval. Adjusted models: Model 1 is adjusted for sex, ethnicity, subjective socioeconomic status, BMI z-score, and age. Model 2 is Model 1 + interaction term between social media use and sex; Model 3 is Model 1 + interaction term between social media use and age; Model 4 is Model 1 + interaction term between social media use and parental support. ^*^p < 0.05; ^**^p < 0.01; ^***^p < 0.001. Bold values represent statistically significant results.

Interactions were statistically significant for age by heavy social media use for distress [Model 3—two-way interaction terms (OR: 0.54; CI: 0.33–0.90, *p* < 0.05)]. Interaction terms for sex (Model 2) and parental support (Model 4) by social media were not significant.

Our unadjusted models show that the interaction terms for parental support by heavy social media for psychological distress are significant for those who reported having parental support sometimes and those who reported rare parental support (OR: 0.51; CI: 0.27–0.94, *p* < 0.05 and OR: 0.53; CI: 0.31–0.91, *p* < 0.05, respectively). However, the moderating role of parental support became insignificant after adjusting for covariates. Thus, subsequent analyses examining the association between social media use and psychological distress were stratified by age.

### Crude and adjusted multivariable ordinal regression modeling of social media use on psychological distress among adolescents stratified by age

Results of the multivariable ordinal regression analysis examining the association between social media use stratified by age are presented in Table 5. After adjusting for covariates, younger students who use social media heavily had higher odds of psychological distress (OR = 2.32; CI: 1.67–3.21).

**Table 5:** Results of the crude and adjusted ordered regression analysis of the associations between social media use and psychological distress among adolescents, stratified by age (*n* = 6,822).

Characteristics	Age	
	11– 14-year-old	15–20-year-old
	*N* = 2,880	*N* = 3,942
Model 1—Crude		
No use	1	1
Light to moderate use (≤ 2 h/day)	1.17 (0.88–1.54)	0.82 (0.59–1.24)
Heavy use (≥ 3 h/day)	**2.50 (1.83–3.42)** ^***^	1.45 (1.01–2.13)^*^
Model 2—Adjusted		
No use	1	1
Light to moderate use (≤ 2 h/day)	1.27 (0.95–1.70)	0.86 (0.58–1.26)
Heavy use (≥ 3 h/day)	**2.32 (1.67–3.21)** ^***^	1.24 (0.86–1.78)

Data are presented as odds ratio and 95% confidence interval. OR, odds ratio; CI, confidence interval.

Model 1 is unadjusted. Model 2 is adjusted for sex, ethnicity, subjective socioeconomic status, and BMI *z*-score.

^*^p < 0.05; ^**^p < 0.01; ^***^p < 0.001. Bold values represent statistically significant results.

## Discussion

This study examined the association between social media use and psychological distress in a large and representative sample of adolescents in Ontario (Canada) and explored whether sex, age, and parental support would moderate this relationship. Heavy use of social media was moderately associated with higher levels of psychological distress among adolescents, and the effect of the association was modified by age, but not sex or parental support.

Even though the cross-sectional design of this study cannot determine the direction of the associations, our findings regarding the positive correlation between time spent on social media and high levels of psychological distress align with previous cross-sectional (42, 63–67), experimental and prospective studies (68–71), and reviews (24, 34, 73, 74), which provided implicit causation regarding the direction of the association that moves from heavy social media use to mental health problems. Numerous mechanisms could contribute to the direction and nature of the relationship between social media use and depression and anxiety. Adolescents involved in high levels of social media may experience poor quality of sleep, which plays a mediating role in the pathway to depression and anxiety (75, 76). Furthermore, time spent on social media may increase the risk of exposure to unrealistic societal ideal images and an over representation of positive experiences and posts (77, 78). Thus, adolescents who make frequent unfavorable social comparisons with other users are more likely to experience negative feelings about body image and encounter envy, guilt, and regret (66, 79). These feelings can be detrimental to their self-esteem and have been identified as significant contributors to internalizing problems such as depression and anxiety (25, 79–83). Also, more time spent on social media increases the chances of experiencing cyberbullying, which has strong ties with depressive symptoms (47, 84). In addition, the displacement theory posits the lack of social and in-person interaction and family communication displaced by frequent time on social media can contribute to symptoms of depression and anxiety (85). These associations between social media and mental distress should be explored prospectively to help determine the temporality of the associations identified.

This study also found that younger adolescents have higher odds of distress when using social media heavily than their older counterparts (42). Our results could be explained by early adolescence, as a vulnerable stage for mental health issues (4, 5, 86, 87) in which social media was indicated as a potential risk factor (88, 89). This phase involves significant pubertal changes, and biological, neurological, and social transformation (5, 90, 91), in which adolescents start to experience an increase in self-consciousness, inner conflict, stress, and disorientation (91). At the social and contextual level, peer relationships become more central to self-worth, and family can become disoriented as youths strive for independence, and this may spark greater reliance on social media as a source of social support, autonomy, seeking peers’ feedback, and exploring identity (43, 92–94). As such, early adolescence is distinguished by a significant increase in internalizing and externalizing problems (4, 5, 86, 87). Young adolescents’ brain regions that are engaged in social interaction endure extensive changes, which make them vulnerable to being impacted by media use that contributes to low self-esteem, upward social comparison, emotion-loaded communications (25, 92), and cyberbullying which can all trigger mental health symptoms (37, 95–97). In addition to that, heavy use of social media can interfere with the time adolescents use to do other beneficial activities like in-person interaction or physical activity which can increase their chance to experience mental health issues (14, 98).

While males were less likely than females to experience psychological distress, and tend to use social media less heavily, no moderating role of sex was found in the extent to which social media use subgroups are associated with psychological distress, aligning with previous work (40). These results may be attributed to the way social media use was measured, which only focused on the social media use time and could not represent how social media was used. However, our study showed that females spent more time on social media and reported greater psychological distress symptoms than males, results in line with previous studies (4, 10, 40, 42, 88, 99–101). We hypothesized that females would be more negatively affected psychologically by greater social media use because they are more emotionally and interested in friendships, and body appearance (102, 103), and tend to be more involved in self-disclosure (102), and are more likely than males to experience low emotional self-efficacy and lack of negative emotional management (104, 105). This might make females more vulnerable to experience social media-related upward social comparison (106), cyberbullying (36, 47), and body dissatisfaction (89), all factors known to be linked with depression and anxiety.

The finding that adjustment for SES and sex reduced the moderating effect of parental support to nonsignificance suggests that parental support’s role in the association between social media use and psychological distress among adolescents is impacted by sex and SES. This supports the need for future longitudinal studies to evaluate further these factors and other unexamined factors that may underlie the moderating effects of parental support in the social media–mental health relationship. In further consideration of our findings, the simplicity of the parental support variable, which only measured to what extent adolescents discuss their problems with parents, could be one reason why parental support did not moderate the association after adjustment for such a finding. Research has indicated that the role of parents is indeed very important and includes multiple levels of involvement, in addition to having their children discuss their problems with them (24, 45). Parents can be both gatekeepers (107) and influencers of their adolescents’ social media use experience especially during early adolescence (108–110).

On the other hand, our results showed that those with rare parental support used social media more frequently and had higher odds for psychological distress, consistent with previous work. It has been known that parents play a significant role in their adolescents’ social media use and behavior, which can impact mental well-being (111–114). Adolescents who reported having unsupportive parents felt disconnected, and used social media more frequently to find meaningful social interaction (45). That being said, in-person communication and support offline from parents are all factors that can shape adolescents’ media use and experience, which in turn impact mental well-being. Therefore, we highly recommend that future research investigate through large longitudinal studies the role of parental support, taking into consideration the impact of other important covariates in this association.

This study has several limitations worth mentioning. Our findings are based on cross-sectional data; thus, the bidirectional relationship between social media use and psychological distress could not be determined. Therefore, it is still unclear whether social media experiences shape mental health problems or vice versa. For example, those in distress may seek comfort, support or satisfy other psychological needs through social media (115). Also, data measured only the time spent on social media and not the quality of the time, which is a critical factor (29, 37). In addition, self-reported instruments are subject to inaccuracies and residual confounding by unmeasured variables is always possible in observational studies (e.g., medication use and history of mental health problems). Finally, as in all large studies, missing values are inevitable, and we had 10%. Case analyses showed that those with missing data were more likely to be males, to use social media more heavily and were more distressed, so it is uncertain how these missing cases would have impacted the observed associations had they been included in the analysis.

The results of our study may offer valuable implications for tailored preventative interventions to help minimize heavy and unhealthy social media use. As our results indicated, younger adolescents are more likely to experience psychological distress when using social media heavily; as such, it is important to educate parents and caregivers to help their young children and adolescentsto decrease their time on social media and promote appropriate behavioral regulation. Also, discussions with adolescents about their social media behavior, the risks and benefits of platforms, and promoting safe and effective social media strategies are warranted (116, 117). Furthermore, longitudinal studies that capture specific types, quality, and context of social media use are highly recommended to better understand the strength and the directional impact of the relationship between social media and mental health.

## Conclusion

Our results show that heavy social media use is moderately associated with psychological distress among adolescents, and this association is modified by age, with younger adolescents being more vulnerable. As such, future studies would benefit from longitudinal designs with larger, more representative samples that examine the bidirectional association and investigate in more depth the role of sex, age, and parental support. Furthermore, future studies that investigate the mechanisms of this association taking into consideration the quality and quantity of social media use, are warranted. Addressing these points will help to design media literacy programs, policies, and approaches for parents, mental health providers, teachers, and adolescents to promote mental health and educate adolescents, especially younger ones, on the negative psychological impacts of heavy social media consumption.

## Data availability statement

The data analyzed in this study is subject to the following licenses/restrictions: the datasets presented in this research article cannot be made available in the manuscript, supplementary material, or a public repository due to the Center for Addiction and Mental Health’s and The Ontario Public and Catholic School Board’s institutional Research Ethics Board agreements. Requests to access the datasets should be directed to the Center for Addiction and Mental Health at info@camh.ca.

## Ethics statement

The studies involving human participants were reviewed and approved by the Research Ethics Boards of the Center for Addiction and Mental Health (CAMH), York University, and 47 public and Catholic school boards’ research review committees. Written informed consent to participate in this study was provided by the participants’ legal guardian/next of kin.

## Author contributions

FM, J-PC, and GG participated in the conception of the study. FM and HS-K conducted statistical analyses. FM wrote the first version of the manuscript. J-PC and GG substantially contributed to the methods and interpretation of results. J-PC, GG, HS-K, IC, SL, and HH critically reviewed the manuscript. All authors contributed to the article and approved the submitted version.

## Funding

The Ontario Student Drug Use and Health Survey, a Center for Addiction and Mental Health initiative, was funded in part through ongoing support from the Ontario Ministry of Health and Long-Term Care, as well as targeted funding from several provincial agencies.

## Conflict of interest

The authors declare that the research was conducted in the absence of any commercial or financial relationships that could be construed as a potential conflict of interest.

## Publisher’s note

All claims expressed in this article are solely those of the authors and do not necessarily represent those of their affiliated organizations, or those of the publisher, the editors and the reviewers. Any product that may be evaluated in this article, or claim that may be made by its manufacturer, is not guaranteed or endorsed by the publisher.
